# Analyses of Impact of Needle Surface Properties on Estimation of Needle Absorption Spectrum: Case Study with Coniferous Needle and Shoot Samples

**DOI:** 10.3390/rs8070563

**Published:** 2016-07-02

**Authors:** Bin Yang, Yuri Knyazikhin, Yi Lin, Kai Yan, Chi Chen, Taejin Park, Sungho Choi, Matti Mõttus, Miina Rautiainen, Ranga B. Myneni, Lei Yan

**Affiliations:** 1 Beijing Key Laboratory of Spatial Information Integration and 3S Application, Institute of RS and GIS, School of Earth and Space Sciences, Peking University, Beijing 100871, China; 2 Department of Earth and Environment, Boston University, Boston, MA 02215, USA; 3 School of Geography, State Key Laboratory of Remote Sensing Science, Beijing Normal University, Beijing 100875, China; 4 Department of Geosciences and Geography, University of Helsinki, P.O. Box 68, Helsinki, FI 00014, Finland; 5 Schools of Engineering and Electrical Engineering, Aalto University, P.O. Box 15800, Aalto 00076, Finland

**Keywords:** leaf albedo, leaf biochemistry, leaf surface reflectance, polarization measurements

## Abstract

Leaf scattering spectrum is the key optical variable that conveys information about leaf absorbing constituents from remote sensing. It cannot be directly measured from space because the radiation scattered from leaves is affected by the 3D canopy structure. In addition, some radiation is specularly reflected at the surface of leaves. This portion of reflected radiation is partly polarized, does not interact with pigments inside the leaf and therefore contains no information about its interior. Very little empirical data are available on the spectral and angular scattering properties of leaf surfaces. Whereas canopy-structure effects are well understood, the impact of the leaf surface reflectance on estimation of leaf absorption spectra remains uncertain. This paper presents empirical and theoretical analyses of angular, spectral, and polarimetric measurements of light reflected by needles and shoots of *Pinus koraiensis* and *Picea koraiensis* species. Our results suggest that ignoring the leaf surface reflected radiation can result in an inaccurate estimation of the leaf absorption spectrum. Polarization measurements may be useful to account for leaf surface effects because radiation reflected from the leaf surface is partly polarized, whereas that from the leaf interior is not.

## 1. Introduction

Leaf level physiological processes are among the climate variables that directly control the dynamic of ecosystems. Quantifying changes in leaf biochemistry provides direct information about ecosystem functioning and a method to detect and monitor changes in response to climate changes [1–3]. The leaf scattering spectrum is the key optical variable that conveys information about leaf level physiological processes from remote sensing. The radiation scattered by leaves and exiting the vegetation canopy is affected by the 3D canopy structure. The leaf scattering properties cannot be estimated from space measurements without accounting for canopy structural effects [4,5]. In addition, characteristics of the leaf surface are important to remote sensing of leaf biochemistry. Some radiation is scattered at the surface of leaves [6–9]. This portion of reflected radiation does not interact with the leaf interior and therefore contains no information about absorbing biochemical constituents inside the leaf. This presents an additional confounding factor, unless it can be accounted for. Very little empirical data are available on the spectral and angular scattering properties of the leaf surface. Whereas canopy-structure effects are well understood [4,10–12], the impact of the leaf surface reflectance on the estimation of leaf absorption spectra and, consequently, concentrations of leaf absorbing biochemical constituents remains uncertain.

**Figure 1:**
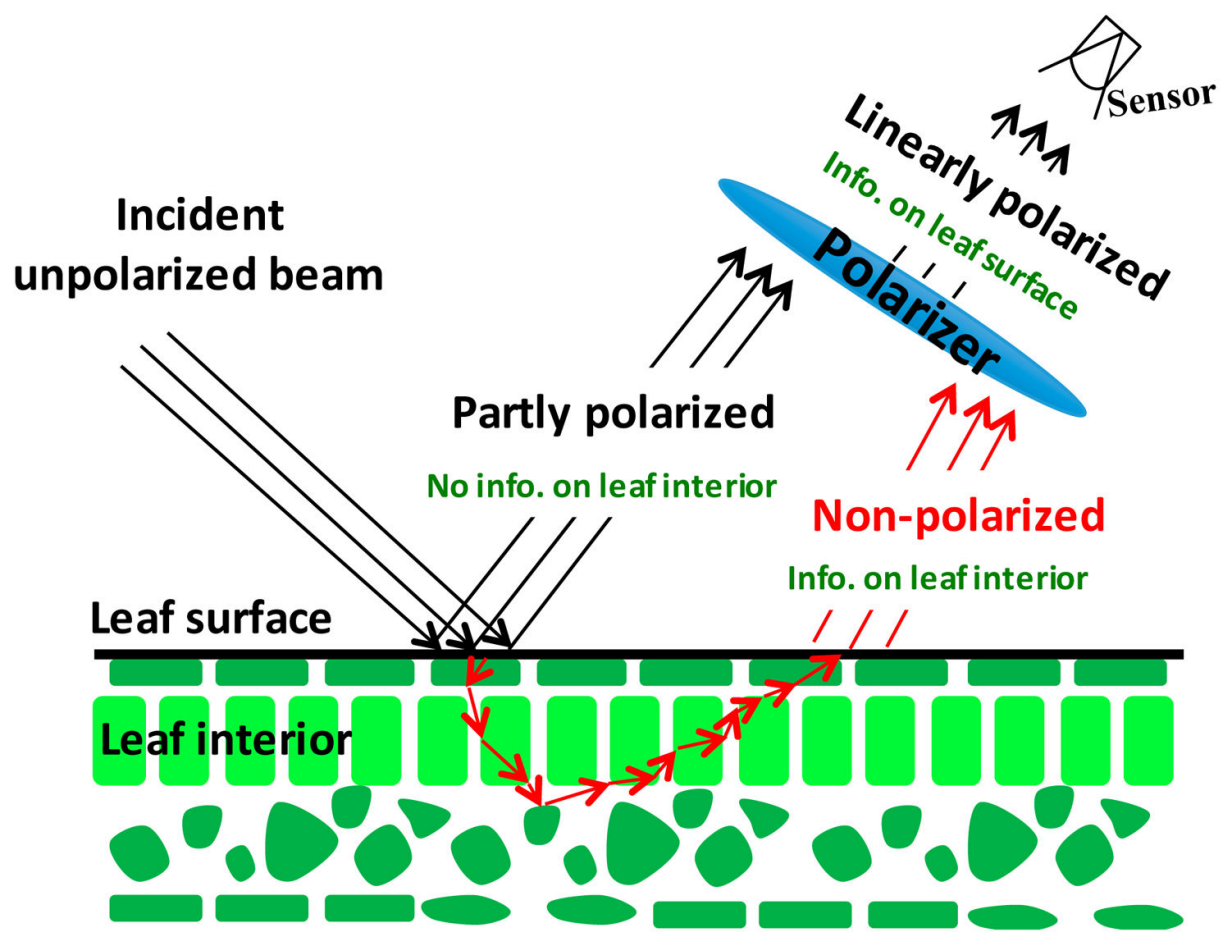
Radiation reflected by a leaf includes two components, specular and diffuse. The first component, emanating from light reflected at the air-cuticle interface is partly polarized. This portion of reflected radiation does not interact with pigments inside the leaf, but depends on the properties of the leaf surface. The diffuse component that mainly results from radiation interactions within the leaf-interior is not polarized. Its spectral behavior depends on the intrinsic optical properties of leaf biochemical constituents.

Solar radiation scattered by a leaf includes two components, specular and diffuse (Figure 1) [13,14]. The first component results from light reflected at the air-cuticle interface. This fraction of reflected radiation is partly polarized [6,7]. Quasi-specular reflection is the primary mechanism that polarizes the reflected light [6–9]. The diffuse component, which results from photon interactions within the leaf and any large particles on the leaf surface, is not polarized. Its spectrum is mainly determined by the absorption properties of leaf biochemical constituents and therefore carries information about their concentrations. Reflectance measurements alone cannot discriminate between radiation scattered from the leaf surface and leaf interior. Polarization measurements can be used to partly separate the specular component from the total reflectance [6,7,9].

This paper presents empirical and theoretical analyses of spectral, angular and polarimetric measurements of light reflected by needles and shoots of two coniferous species. The objective of this paper is to quantitatively and qualitatively describe the process of photon interactions with needles and shoots, with an emphasis on understanding the impact the leaf surface properties might have on estimation of leaf absorption spectrum.

The paper is organized as follows. A description of our study area, samples, the setup of laboratory measurements and data processing approaches are given in Section 2, and Appendices A and B. Empirical and theoretical analyses of the measured directional conical reflectance factor (DCRF) data are presented in Sections 3 and 4, respectively. Finally, Section 5 summarizes the results.

## 2. Materials and Methods

### 2.1. Samples

Shoots from mature *Pinus koraiensis* and *Picea koraiensis* trees were collected in campus of Northeast Normal University, Changchun, China (N 43.88, E 125.35) on 9 March (*Pinus koraiensis*) and 10 March (*Picea koraiensis*), 2013. Changchun has a temperate monsoon climate (annual mean temperature 4.8 °C and precipitation 570 mm). *Pinus koraiensis* and *Picea koraiensis* are typical species in this area [15,16]. The exposed shoot samples (one from *Pinus koraiensis* and another one from *Picea koraiensis*) were taken from bottom parts of the crowns. Each sample consisted of two sister shoots (same-year, same-structure and growing in same environment). From the sister shoots, one shoot was used for measuring needle optical properties and the other shoot for measuring shoot optical properties. The samples were stored in zip-locked plastic bags. Needle and shoot optical properties were measured about 2 h after sampling in laboratory conditions. These data underlie our analyses of physical mechanisms of photon interactions at needle and shoot scales.

**Figure 2:**
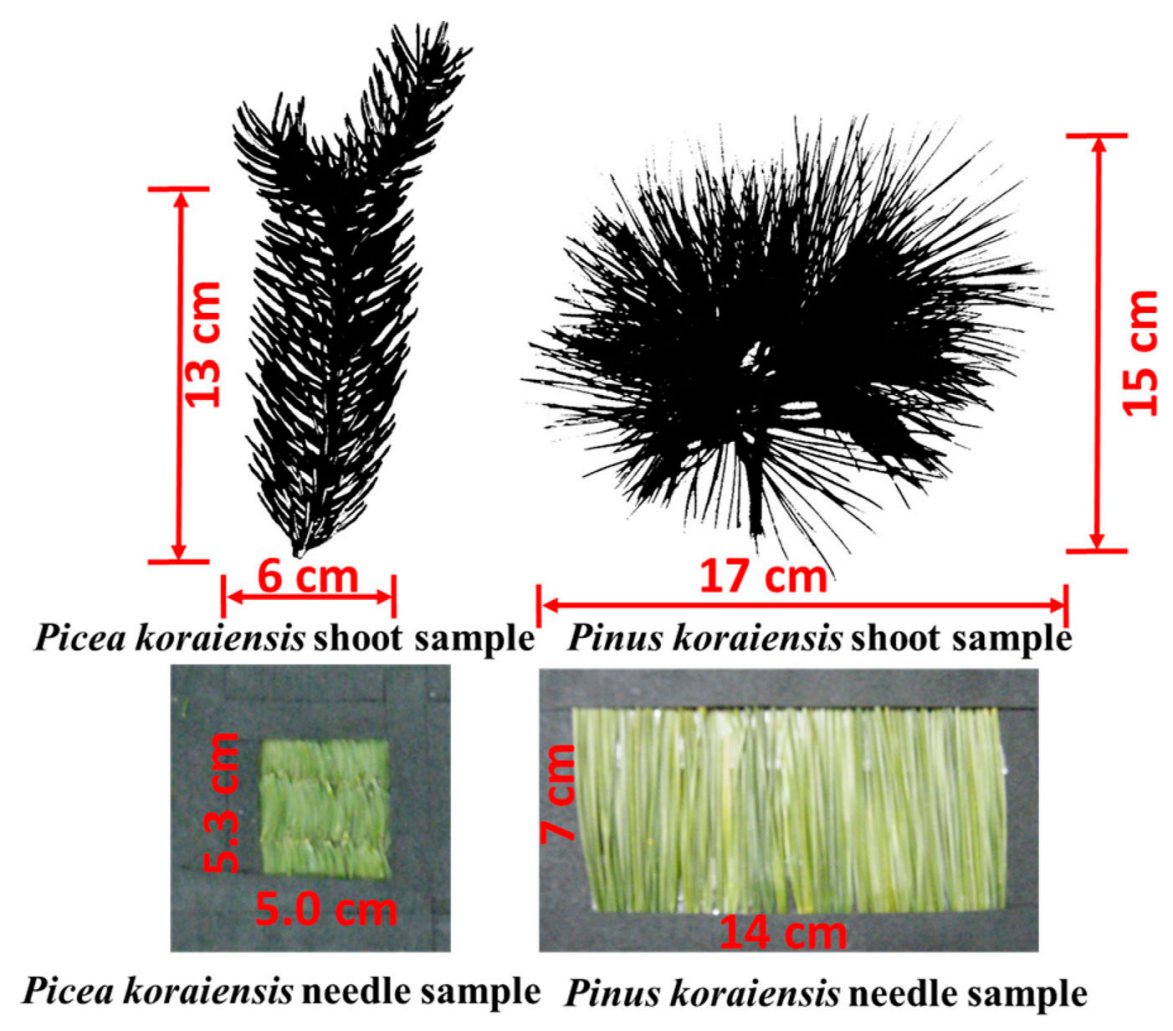
Samples of shoots and needles in the holder window. Sizes of the shoots were 13 cm by 6 cm (*Picea koraiensis*) and 15 cm by 17 cm (*Pinus koraiensis*). Dimensions of the holder windows were 5.3 cm by 5.0 cm (*Picea koraiensis*) and 7 cm by 14 cm (*Pinus koraiensis*).

A *Picea koraiensis* sample consisting of two sister shoots about 13 cm in twig length and 6 cm in diameter was selected for measurements. The numbers of needles in the sister shoots were 150 and 167, respectively. Mean needle length of the sample was 2.1 (±0.6) cm. The needles were rhombic in cross-section and covered the whole twig [15]. Twig lengths of *Pinus koraiensis* sister shoots were 15 and 17 cm. Numbers of needles in the shoots were 95 and 103, respectively; mean needle length of the sample was 9.0 (±1.3) cm. The needles were awl-like, almost triangular in shape cross-sections and located on the twig in bundles of five [16]. To measure needle DCRF, the needles were placed parallel to each other in a holder window and secured using black tape (Figure 2). The holder was covered by the black tape. The nadir DCRF spectrum of the black tape was around 5% in the interval 450–950 nm. The needles in the holder window formed rough surfaces. The holder window dimensions were 5.3 cm by 5.0 cm (*Picea koraiensis*) and 7 cm by 14 cm (*Pinus koraiensis*). To measure shoot DCRF, the shoots were placed on a black-tape board.

### 2.2. Instrumentation

**Figure 3:**
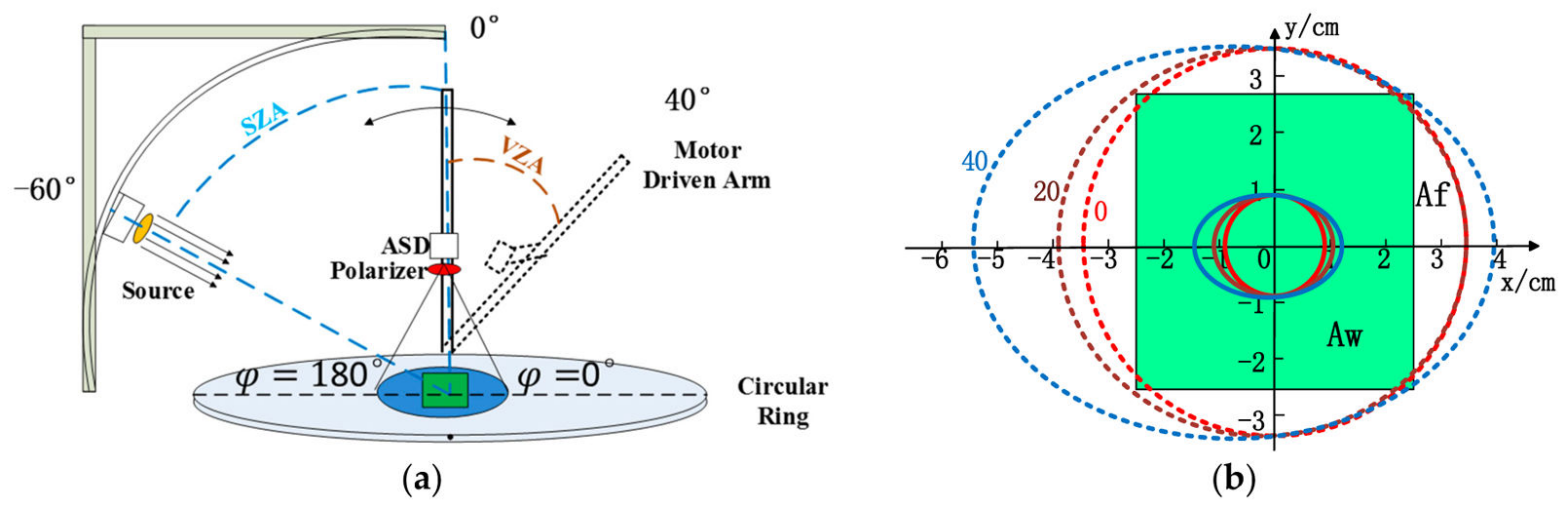
(**a**) The goniometer system. The system includes a source, a circular ring and a motor driven arm. The circular ring and the motor driven arm can provide any source-sensor configurations in the upper hemisphere; (**b**) Footprints of the Analytical Spectral Devices FieldSpec 3 (ASD FS3) with field of view (FOV) = 20° (dashed lines) and FOV = 6.08° (solid lines) for view zenith angles (VZAs) of 0°, 20° and 40°. The origin corresponds to the center of the circular ring. The green rectangle depicts the holder window of the area *A_w_* = 5.3 cm × 5.0 cm with *Picea koraiensis* needles. The area outside the holder window is a black surface (directional conical reflectance factor (DCRF) = ~5%).

DCRFs of needle and shoot samples were measured by a spectroradiometer, which was mounted on a goniometer system designed by Yunsheng Zhao [17]. The system consisted of a goniometer, a source of light, an Analytical Spectral Devices FieldSpec 3 (ASD FS3) spectroradiometer (Analytical Spectral Devices, Inc., Boulder, CO, USA) and a Glan–Thomson polarizer (Figure 3a) (Chinese Academy of Sciences, Hefei, Anhui, China).

The goniometer has a 1.2 m long motor driven arm mounted on a circular ring. The sample was positioned in the center of the circular ring. The distance between the sample material and the sensor was set to 19.8 cm. View and source zenith angles have an accuracy of 0.1° and 0.25°, respectively, and the relative azimuth angles between the source and sensor have an accuracy of 0.25°.

The illumination source was a 500 W tungsten halogen lamp, which was collimated using parabolic convex mirrors [17]. The temporal variation of output radiation was less than 2%. The diameter of the beam at source was about 9 cm. The footprint of the beam on the circular ring was an ellipse with major axis 18 cm and minor axis 9 cm.

The ASD FS3 spectroradiometer covered the spectral range from 350 to 1000 nm with spectral resolution of 3 nm at 700 nm. The spectral data were averaged over 10 nm spectral intervals. The instrument was equipped with bare-fiber optics, which had a 25° field of view (FOV) [18]. To decrease the impact of stray light, a view limiting tube was attached to the fiber, restricting its FOV to 20°. The ASD was used in radiance mode. The integration time was set to 136 ms. Five spectra were measured at each measurement angle and their average was recorded by the ASD.

To measure degree of linear polarization (DOLP), a Glan–Thomson polarizer with FOV = 6.08° was attached to the ASD fiber [17]. The polarizer’s optical axis could manually be rotated from *ψ* = 0° to *ψ* = 360° in steps of 1°. Here, *ψ* is the direction of the polarizer’s optical axis; *ψ* = 0° was calibrated as the direction in the plane perpendicular to the view direction as the transmitted radiance approached its maximum. The polarizer transmitted electromagnetic wave with electric field parallel to the polarizer’s optical axis *ψ*, which was then recorded by the spectroradiometer. The transmitted light, *Ī* (*ψ*), can be expressed in terms of the Stokes parameters *I*, *Q* and *U* as *Ī* (*ψ*) = 0.5(*I + Q*cos*ψ* + *U*sin2*ψ*)[19]. We measured the radiance *Ī* (*ψ*) for *ψ* = 0°, 60° and 120°. The DOLP was estimated from these measurements as:

(1) $$\mathrm{DOLP}=\frac{\sqrt{Q^{2}+U^{2}}}{I},$$

where the Stokes parameters were calculated as

(2) $$I=\frac{2}{3}\;\left[\bar{I}(0{}^{\circ})+\bar{I}(60{}^{\circ})+\bar{I}(120{}^{\circ})\right],$$

(3) $$Q=\frac{2}{3}\;\left[2\bar{I}(0{}^{\circ})-\bar{I}(60{}^{\circ})-\bar{I}(120{}^{\circ})\right],$$

(4) $$U=\frac{2}{\sqrt{3}}\;\left[\bar{I}(60{}^{\circ})+\bar{I}(120{}^{\circ})\right].$$

The Stokes *V* component that describes the elliptical polarization of the light is negligible in the case of vegetation because of the inherent randomness of the properties of the vegetation [20,21] and we thus did not measure this parameter.

### 2.3. Measurements

All DCRF measurements were performed in the principal plane. In our coordinate system, we assigned the sign “minus” to zenith angles for back- and “plus” for forward scattering directions (Figure 3a). Samples were placed in the center of the circular ring (Figure 3a). Needles in the holder were perpendicularly-aligned with the principal plane. Shoot twigs were aligned with the principal plane. The source zenith angle (SZA) was set to −60°. The view zenith angle (VZA) was sampled at equal steps of 10° from −30° to +40°. To minimize effects of diffuse illumination, windows of the laboratory were covered with black curtains. The floor inside the goniometer was covered by black paint. We followed the following measurement protocol to obtained data needed to estimate spectral DCRF, Stokes parameters and DOLP of a sample:

1. Perform ASD FS3 measurements of signals reflected by a calibrated 30 cm by 30 cm Spectralon white reference panel (Chinese Academy of Sciences, Anhui, China) in all view directions.
2. Place a sample in the center of the circular ring and perform ASD FS3 measurements of the reflected signals in all view directions.
3. Attach the Glan–Thomson polarizer and measure reflected polarized radiance *Ī* (*ψ*) for *ψ* = 0°, 60°, 120°, in all view directions.

It took about 1 h to accomplish measurements of one sample.

### 2.4. Data Processing

#### 2.4.1. Correction for Footprint Effects

The DCRF is the ratio of the reflected conical irradiance from the surface area *A* to the reflected conical irradiance from an ideal and diffuse surface of the same area *A* under identical source and view geometry [22]. In our measurements, however, the area, *A_w_*, of the holder window with needles and the *Picea koraiensis* shoot were within the footprint area *A_f_* (Ω) of the ASD FS3 with FOV = 20 in all viewing directions (Figure 3b). A black surface outside the holder window was also in the sensor FOV. This discrepancy results in an underestimation of the DCRF. To correct the measured DCRF for the footprint effect, a correction coefficient, *k* (Ω), defined as the ratio of reflected conical irradiance from the Spectralon panel to the reflected conical irradiance from the Spectralon surface area of *S* (Ω) = *A_w_* ∩ *A_f_* (Ω) was calculated (Appendix A). The DCRF was estimated as:

(5) $$\text{DCRF}\;(\Omega_{s},\Omega )=k\;(\Omega )\;\frac{{\Phi^{\prime }}_{s}\;(\Omega_{s},\Omega )}{\Phi_{b}\;(\Omega_{s},\Omega )},$$

where Φ′*_s_* and Φ*_b_* represent ASD FS3 measurements of our sample and the Spectralon white reference panel (Steps 1 and 2 in Section 2.3), respectively; Ω*_s_* and Ω denote directions to the source and sensor, respectively. Values of the correction coefficient are given in Table 1.

#### 2.4.2. Inter-Calibration of ASD FS3 and Polarizer

The difference in FOV of the ASD FS3 (FOV = 20°) and Glan–Thomson polarizer (FOV = 6.08°) can result in different estimates of DCRF of the same heterogeneous target. To account for this effect, an inter-calibration coefficient defined as:

(6) $$c_{\lambda }\;(\mathrm{VZA},\text{sample})=\frac{I_{P}}{I}\frac{L\;(\text{FOV}=20{}^{\circ})}{L\;(\text{FOV}=6.08{}^{\circ})},$$

was calculated for each sample. Here, *I_P_* (Equation (2)) and *I* represent radiances of a sample reflected radiation from ASD FS3 measurements with and without polarizer, respectively; *L* (FOV) is the reflected radiance from the Spectralon panel surface of the area *S* (Ω) registered by the sensor with a given FOV. For each sample, the coefficient was derived for three spectral intervals that represent photosynthetically active radiation (450–640 nm), red edge (650–690 nm) and near infrared (700–950 nm) wavelengths. For each spectral interval, VZA and sample, the ratio *I_P_*/*I* was calculated as the slope of the spectral *I_P_* versus spectral *I* regression line. The coefficients of determination exceeded 0.99, and the intercepts were negligibly small for all VZA and samples. Values of *L* (FOV) were calculated using the algorithm described in Appendix A. The DCRF estimated using Equation (5) was multiplied by the calibration coefficient. The VZA-average coefficients are shown in Table 2.

#### 2.4.3. Decomposition

The DOLP was estimated from the radiance data measured with the polarizer (Step 3 in Section 2.3) using Equations (1)–(4). Since the estimation of DOLP does not use the reference Spectralon data, the correction for footprint effects was not performed. To reduce the noise presented in the data, a standard Savitzky–Golay filter [23] (with polynomial order 3 and window size 9) was applied in the spectral space to DCRF and DOLP data separately for each view direction. Given DOLP, the DCRF was decomposed into polarized (PDCRF) and diffuse (DDCRF) components, i.e., DCRF = PDCRF + DDCRF where

(7) $$\text{PDCRF}\;(\Omega_{s},\Omega )=\text{DCRF}\;(\Omega_{s},\Omega )\cdot \text{DOLP}\;(\Omega_{s},\Omega ),$$

(8) $$\text{DDCRF}\;(\Omega_{s},\Omega )=\text{DCRF}\;(\Omega_{s},\Omega )-\text{PDCRF}\;(\Omega_{s},\Omega ).$$

Figure 1 illustrates physical meaning of the decomposition and information contents of its components.

#### 2.4.4. Correction for Sample Structure Effects

The structure of our samples (Figure 2) impacts the measured DCRFs. We use the directional area scattering factor (DASF) to partly correct DCRF data for structural influences. This wavelength-independent variable was originally introduced as a canopy bidirectional reflectance factor (BRF) if the foliage does not absorb radiation [4,24]. The BRF to DASF ratio suppresses the sensitivity of BRF to canopy structure and results in a canopy scattering coefficient, *W_λ_*, defined as the fraction of intercepted radiation that has been reflected from, or diffusively transmitted through, the vegetation. For vegetation canopies with a dark background, the DASF can be directly retrieved from the BRF spectrum in the 710–790 nm interval. We adapted this approach for DDCRF.

The DASF approach uses the concept of the transformed leaf albedo, *ω_λ_*, defined as the fraction of radiation scattered from the leaf interior given that it interacts with internal leaf constituents [25]. In Figure 1, this variable corresponds to the radiation scattered from the leaf interior integrated over all scattering directions. The total fraction of radiation, *ω_λ_*, reflected or transmitted by a leaf (leaf albedo, or single scattering albedo) results from photon interactions with leaf surface and its interior, i.e., *ω_λ_* = *s_L_* + *i_L_ω_λ_*. Here, *s_L_* is the fraction of surface reflected radiation, and *i_L_* = 1 – *s_L_* represents the fraction that enters the leaf interior. This equation is similar to the decomposition shown in Figure 1, with the difference that it relates to spherically integrated variables.

The canopy spectral invariant relationships suggest that the ratio DDCRF*_λ_*/*ω_λ_* is linearly related to DDCRF*_λ_*, i.e., DDCRF*_λ_*/*ω_λ_*= *p*DDCRF*_λ_+ R*, where the intercept *R* and slope *p* are the spectrally invariant directional escape and recollision probabilities [4,24]. Solving this relationship for DDCRF*_λ_*, one obtains:

(9) $${\text{DDCRF}}_{\lambda }\;(\Omega_{s},\Omega )=\frac{R\;(\Omega_{s},\Omega )}{1-\omega_{\lambda }p}\omega_{\lambda }.$$

If needles in our sample do not absorb radiation, i.e., *ω_λ_*= 1, the DDCRF becomes DASF, which is the ratio of the intercept *R* and (1 – *p*). Thus, the DASF can be retrieved from spectral DDCRF if *ω_λ_* at two or more wavelengths is known.

The following result allows the derivation of DASF without prior knowledge of the leaf scattering properties: in the 710–790 nm spectral interval transformed albedos of any two leaves, *ω_λ_* and *ω*_0_*_λ_*, are related via the spectral invariant relationship (Appendix B), i.e.,

(10) $$\omega_{\lambda }=\frac{1-p_{L}}{1-p_{L}\omega_{0\lambda }}\omega_{0\lambda }.$$

Here, *p_L_* is a wavelength independent parameter, which depends on internal leaf constituents. Any transformed leaf albedo, *ω_λ_*, in this spectral interval, therefore, can be standardized to a single known spectrum *ω*_0_*_λ_*, called the reference leaf albedo. By substituting Equation (10) into Equation (9), one obtains that the DDCRF*_λ_* (Ω*_s_*, Ω) in the interval 710–790 nm can be expressed via Equation (9) in terms of either actual albedo *ω_λ_* and spectral invariants *p* and *R*, or the known reference albedo *ω*_0_*_λ_* and the spectral invariants transformed to new values *p*_0_ = *p_L_* + (1 – *p_L_*) *p* and *R*_0_ = (1 – *p_L_*) *R*. It means that DDCRF*_λ_*/*ω*_0_*_λ_* is also linearly related to DDCRF*_λ_* with the slope and intercept given by *p*_0_ and *R*_0_. This does not impact the *R* to (1 – *p*) ratio, i.e., *R*/(1 – *p*) = *R*_0_/(1 – *p*_0_). Thus, the DASF can be estimated from the DDRF spectrum in the 710–790 nm interval using the known reference leaf albedo *ω*_0_*_λ_*. A step-by-step procedure to derive DASF using the reference leaf albedo is documented in [4]. More details about this transformation and its physical interpretation can be found in [24–28].

The reference leaf albedo was specified using Lewis and Disney’s [25] approximation of the PROSPECT model [29,30] with the following parameters: chlorophyll content of 16 *μ*g · cm^−2^, equivalent water thickness of 0.005 cm^−1^, and dry matter content of 0.002 g· cm^−2^ [4].

The scattering coefficient, *W_λ_*, is defined as

(11) $$W_{\lambda }=\frac{{\text{DDCRF}}_{\lambda }\;(\Omega_{s},\Omega )}{\text{DASF}\;(\Omega_{s},\Omega )}=\frac{1-p}{1-\omega_{\lambda }p}\omega_{\lambda }.$$

It depends on the total escape probability, (1 – *p*), which is spherically integrated DASF. Spherical integration significantly lowers the sensitivity of DASF to canopy structure but does not eliminate its impact. The scattering coefficient, therefore, represents DDCRF partly corrected for sample structure effects. Given W*_λ_*, the sample absorption coefficient is *A_λ_*= 1 – W*_λ_*[31], which, in turn, is directly related to the leaf absorption spectrum *a_λ_*= 1 – *ω_λ_* as *A_λ_* = *a_λ_*/[1 – *p* (1 – *a_λ_*)]. Note that our approach allows us to obtain W*_λ_*, but not the recollision probability *p* and transformed albedo *ω_λ_* of a needle. In the case of needles in a shoot or arranged as a horizontal mat, the recollision probability is the probability that a photon scattered by a needle in the sample will interact with a needle again. For a flat leaf, its value is zero and the scattering coefficient coincides with the transformed leaf albedo. For needles in the holder, the recollision probability *p* > 0. A different value can be obtained for needles arranged as the shoot. A relationship between the recollision probability and the shoot structure is discussed in [28].

#### 2.4.5. Sources of Uncertainties

The peak of the source irradiance was located at 700 nm. At wavelengths below 450 nm and above 950 nm, the signal-to-noise ratio was very low. Therefore, we restricted our analyses to the spectral interval 450–950 nm.

Accuracy in aligning the centers of samples and the circular ring was about 0.2 cm. This caused uncertainty in the correction coefficient *k* (Ω). The relative uncertainty increased with VZA and reached its maximum value 1.97% at VZA = 40°.

The anisotropy factor of the reference Spectralon panel exhibited a slow increase from 1 to 1.124 in the interval between zenith and VZA = 40°. For VZA < −30°, the light source was partly blocked by the ASD foreoptics. Therefore, we restricted our analyses of DCRF and its components to VZAs between −30° and +40°. The inter-band difference in the radiance reflected from the Spectralon panel was less than 0.8%, which is acceptable for spectral measurements of surface DCRF [32].

## 3. Results

Radiation reflected by our samples results from photon interactions with the needle surfaces and their interiors (Figure 1, Equation (8)), i.e.,

(12) $${\text{DCRF}}_{\lambda }\;(S_{L})=S_{L}+(1-S_{L})\cdot {\mathrm{DASF}}_{0}\cdot W_{\lambda }.$$

**Figure 4:**
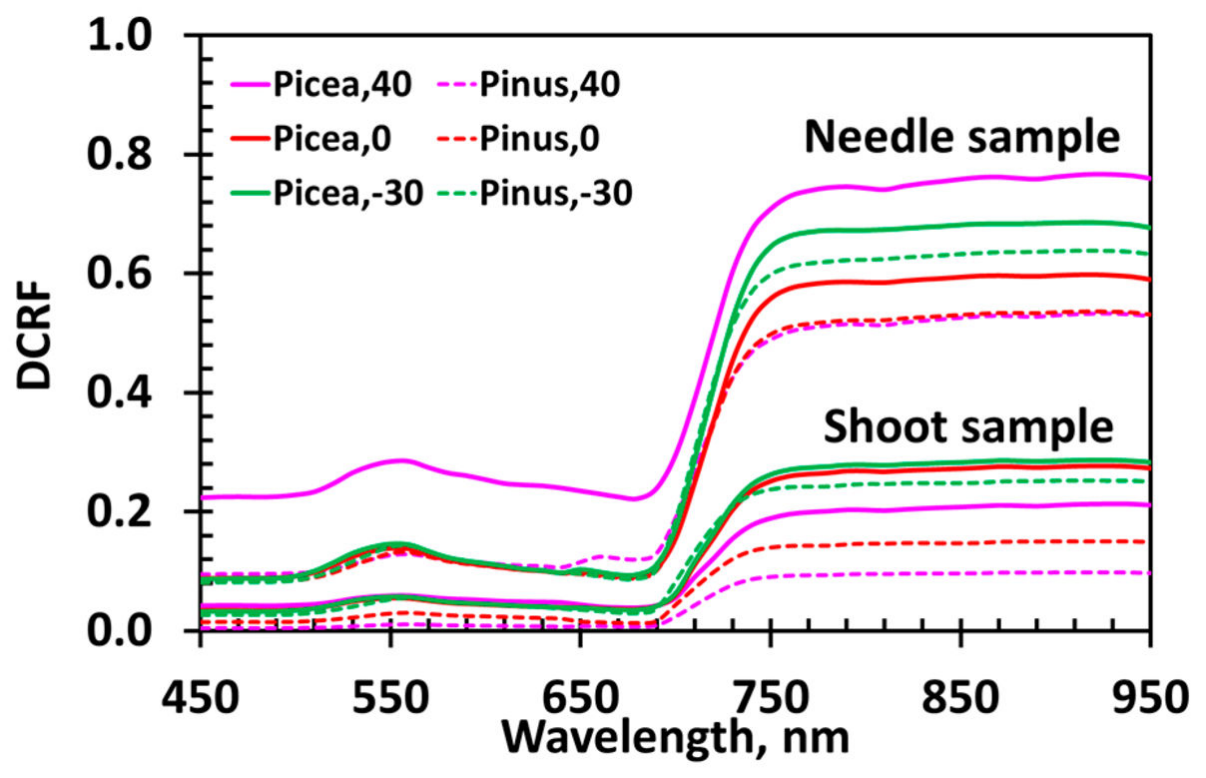
DCRF of *Picea koraiensis* (solid lines) and *Pinus koraiensis* (dashed lines) needle samples and shoots in the spectral interval 450–950 nm for VZA = −30°, 0° and 40°.

Here, *S_L_* represents the component of the measured DCRF due to radiation reflected from the needle surfaces; it does not interact with pigments inside the needles, but depends on the properties of their surfaces. The second term on the right-hand side of Equation (12) is the fraction of radiation reflected from needle interiors in the direction of the sensor. The spectrum of the scattering coefficient *W_λ_* is mainly determined by absorption spectra of absorbing biochemical constituents inside the needles. The radiation exiting the needle interior in the direction of our spectroradiometer is affected by structural properties of the samples. We use a normalized directional area scattering factor, *DASF*_0_ = *DASF*/(1 – *S_L_*), to parameterize the sample structure (Section 2.4.4). The term *DASF*_0_ · *W_λ_* represents diffusely reflected radiation in the absence of scattering at the needle surface. We use *DCRF*_0_ to specify this term, i.e., *DCRF*_0_ = *DASF*_0_ · *W_λ_*. Here, we assume that the contribution of the multiply scattered radiation to the polarized portion of reflected radiation is negligible, i.e., the quasi-specular reflection of the direct incident beam is the only mechanism that polarizes light. This model is based on a solution of the radiative transfer equation, which can be expressed in terms of Equation (12) [4,24]. This equation underlies our analyses of the impact of *S_L_* on the estimation of the scattering coefficient *W_λ_* from DCRF. Figure 4 shows the measured DCRF spectra. The spectral curves vary with samples and VZA.

Our measurements provide an estimate of the lower bound on *S_L_*, i.e., *S_L_* ≥ DOLP*_λ_* · DCRF*_λ_*. We assume that the measured polarized DCRF coincides with the surface reflected radiation *S_L_* and estimate its impact on retrieving *W_λ_* from the measured DCRF under this assumption. As it follows from Equation (12), for a given DCRF, the *DASF*_0_ · *W_λ_* is a decreasing function with respect to *S_L_*. It means that the impact of the surface reflected radiation is stronger than our analysis suggests as long as the *DASF*_0_ is weakly sensitive to *S_L_*.

**Figure 5:**
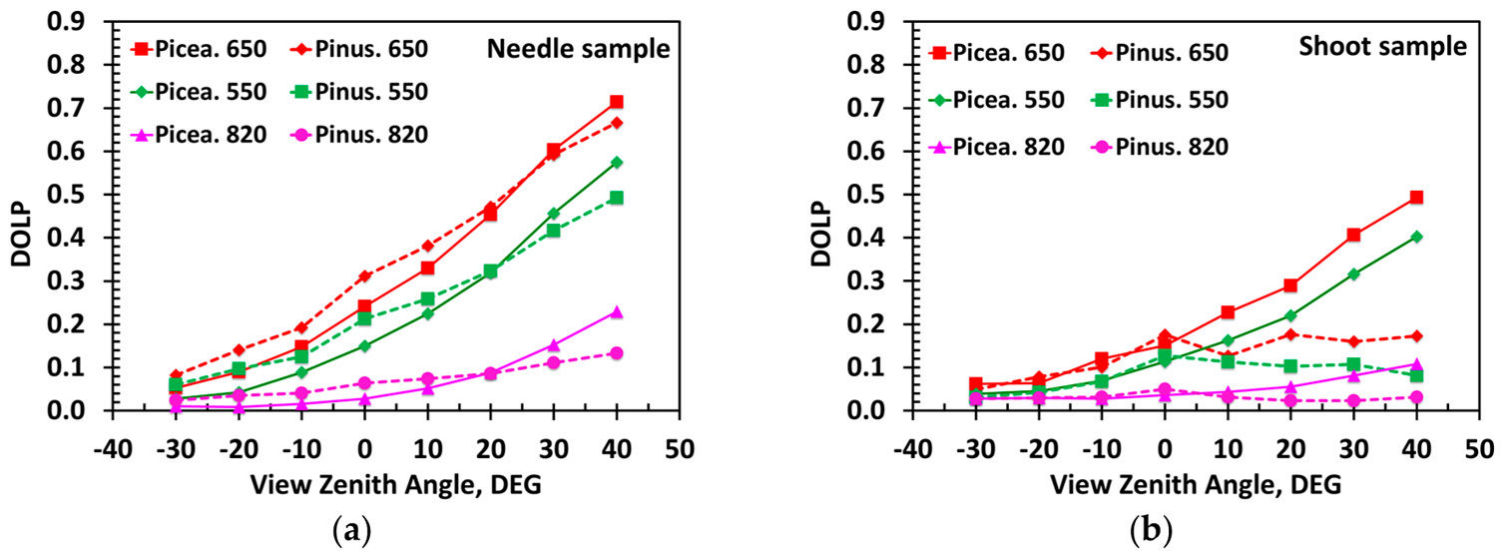
Degree of linear polarization (DOLP) of *Picea koraiensis* (solid lines) and *Pinus koraiensis* (dashed lines) needles (**a**) and shoots (**b**) at green (550 nm), red (650 nm) and near infrared (820 nm) as a function of VZA.

Figure 5 shows DOLP of needle and shoot samples. Radiation specularly reflected by *Picea koraiensis* and *Pinus koraiensis* needle samples exhibit similar tendencies: DOLP increases from back- to forward scattering directions, and decreases from strongly (650 nm) to weakly (820 nm) absorbing wavelengths. Its contribution to the radiation reflected near specular directions (VZA of ~40°) varies between 71% at 650 nm and 13% at 820 nm. DOLP of shoots also follows these regularities although their magnitudes are reduced.

**Figure 6:**
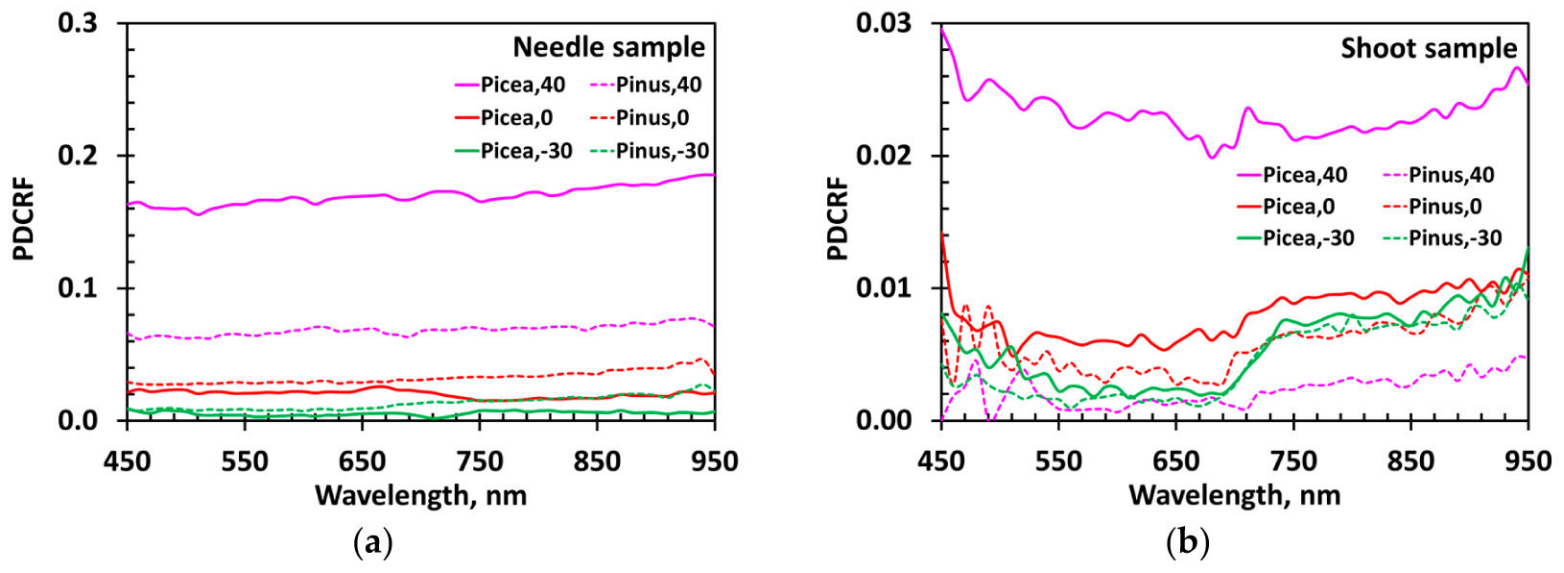
Polarized directional conical reflectance factor (PDCRF) of *Picea koraiensis* (solid lines) and *Pinus koraiensis* (dashed lines) needle samples (**a**) and shoots (**b**) in the spectral interval 450–950 nm for VZA = −30°, 0° and 40°.

**Figure 7:**
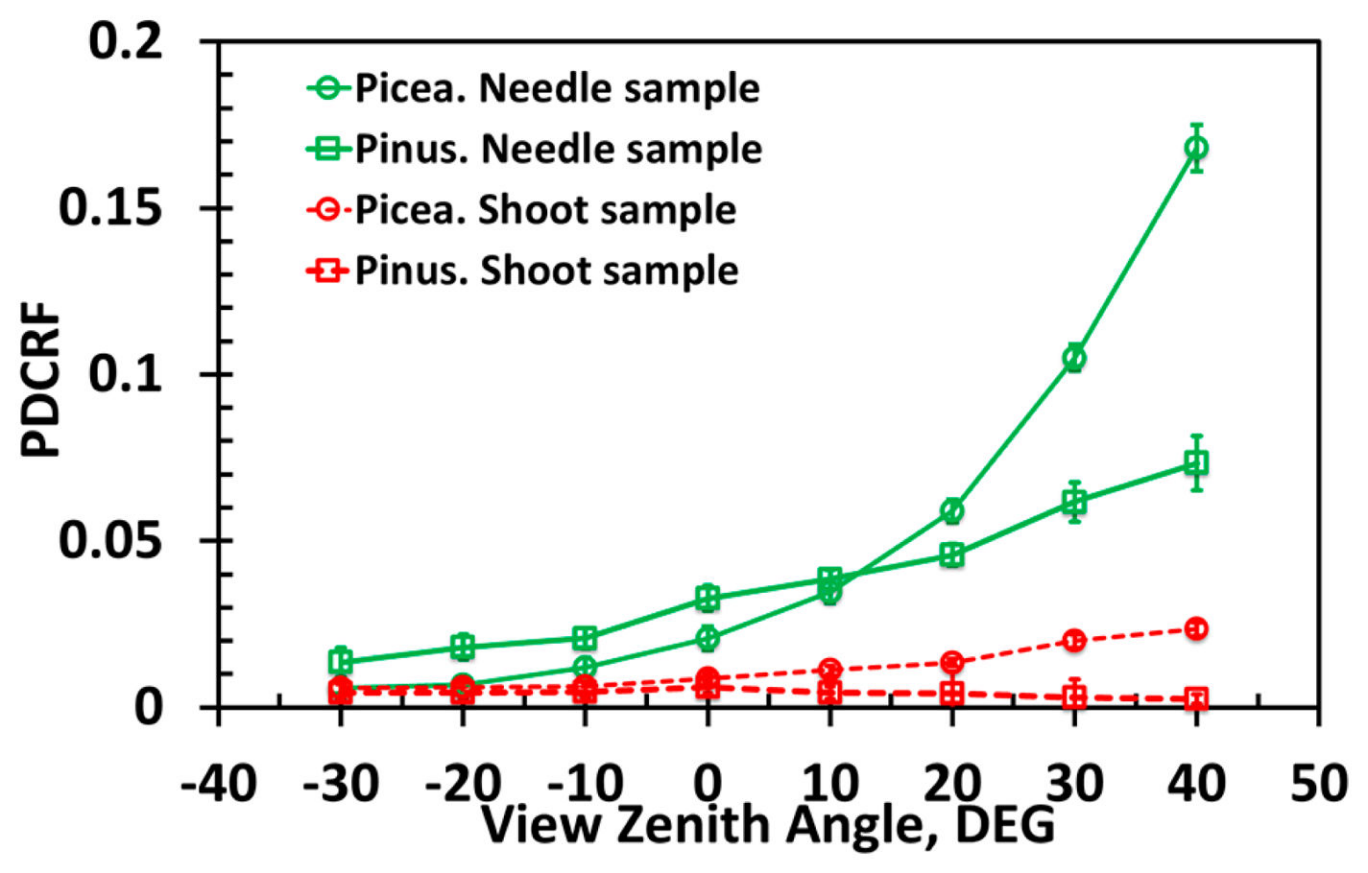
Angular distribution of average PDCRF of needle samples (solid lines) and shoot samples (dashed lines) averaged over 450–950 nm. Vertical bars denote ±1 standard deviation.

Radiation specularly reflected from the needle samples exhibits a weak spectral dependency (Figure 6a), as expected from the theory [9]. Their PDCRF increases from almost negligible values in backscattering directions (VZA < 0°) to about 0.17 when VZA = 40° (Figures 6a and 7). The PDCRF of the shoot reflected radiation displays similar behavior (Figures 6b and 7). However its magnitude is reduced by a factor of about 10. Note that the weak spectral dependency of the surface reflected radiation *S_L_* explains the DOLP spectral behavior (Figure 5): the contribution of the specularly reflected radiation to the total reflected radiation is small when diffuse component is large, as in the near infrared region, and is large when the diffuse component is small, as in the pigment-absorbing blue and red spectral bands.

**Figure 8:**
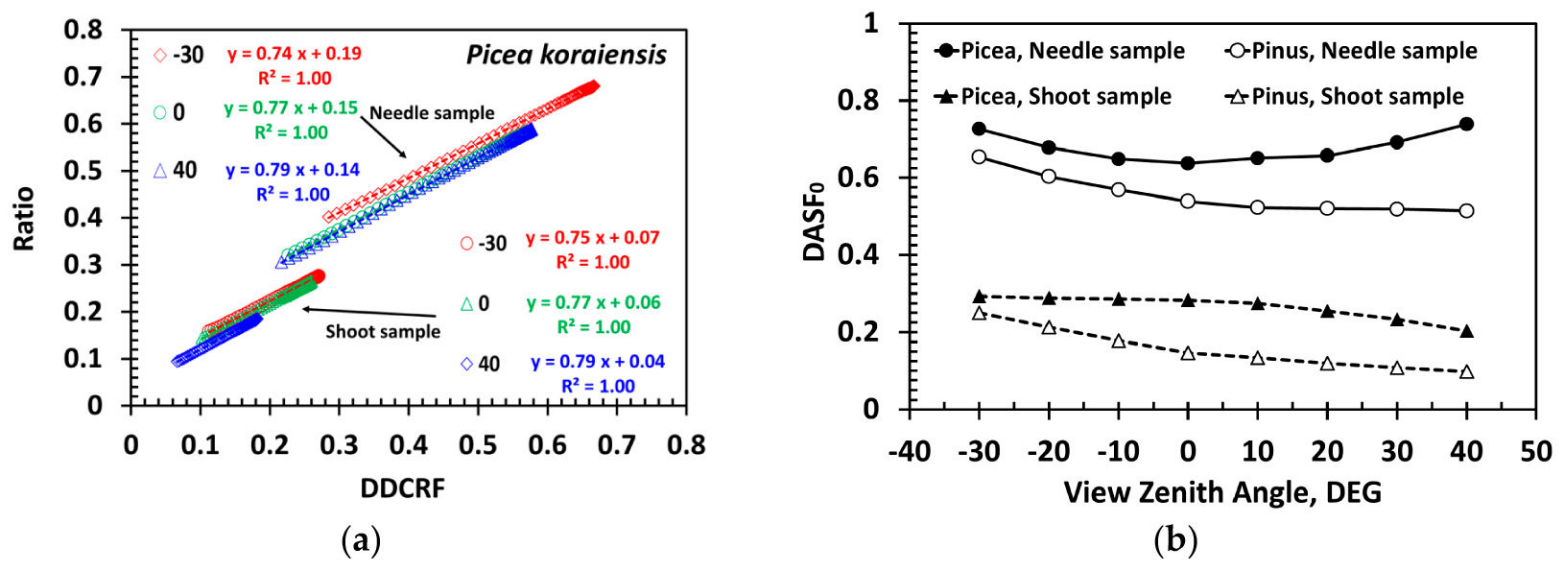
(**a**) Linear relationship between the ratio DDCRF*_λ_*/*ω*_0_*_λ_* and DDCRF*_λ_* for the *Picea koraiensis* samples for VZA = −30°, 0° and 40°. The directional area scattering factor (DASF) is the ratio between the intercept, *R*_0_, and (1 – *p*_0_), where *p*_0_ is the slope (Section 2.4.4). The *Pinus koraiensis* samples follow similar relationship with *R*^2^ = 0.9999 (not shown); (**b**) DASF normalized by (1 – *S_L_*) of needle (solid lines) and shoot (dashed lines) samples.

Figure 8a illustrates that the ratio DDCRF*_λ_*/*ω*_0_*_λ_* is linearly related to DDCRF*_λ_*, as the theory predicts (Section 2.4.4). The normalized *DASF*_0_ is related to the fraction of the (projected) foliage area visible along the viewing direction [4]. Figure 8b shows that *DASF*_0_ of the needle samples is significantly greater than *DASF*_0_ of shoots as expected. Indeed, needles arranged as a horizontal mat in the window holder can intercept and therefore reflect more radiation compared to their shoot counterparts (cf. Figure 7).

**Figure 9:**
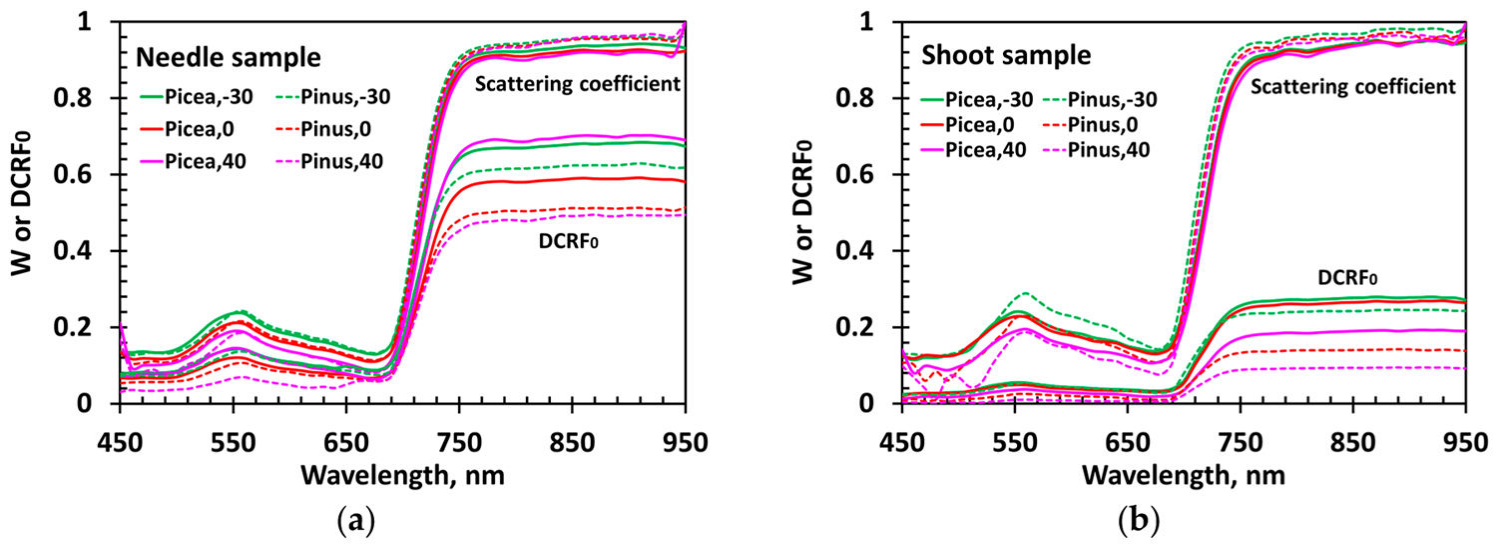
Scattering coefficient W*_λ_* and *DCRF*_0_*_λ_* of *Picea koraiensis* (solid lines) and *Pinus koraiensis* (dashed lines) needle samples (**a**) and shoots (**b**) samples in the spectral interval 450–950 nm for VZA = −30°, 0° and 40°.

**Figure 10:**
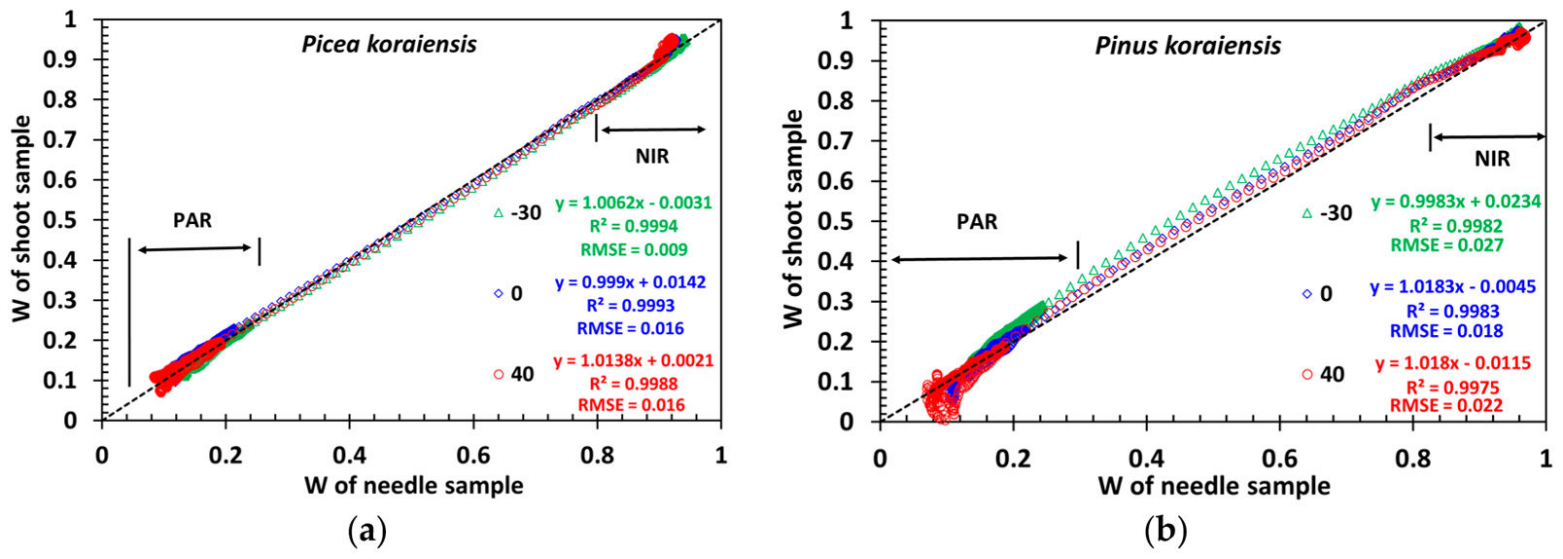
Correlation between needle sample and shoot scattering coefficients of *Picea koraiensis* (**a**) and *Pinus koraiensis* (**b**) for VZA=−30°, 0° and 40°.

Figure 9 shows spectra of DCRF_0_*_λ_*= *DASF*_0_ · *W_λ_* and *W_λ_*. One can see that a decrease in the effect of structural influences involves changes in both the magnitude of the spectral curves and their positions relative to each other. This also significantly lowers angular variation in the structure corrected DCRF. The residual angular variations are due to an incomplete removal of the needle surface reflected radiation *S_L_* from the measured DCRF (Equation (12)). The scattering coefficient replicates the shape and magnitude of typical needle albedo documented in literature [33,34]. Figure 10 shows that the scattering coefficients of needle and shoot samples are very close, suggesting that *W_λ_* is an approximation of the needle albedo. According to Equation (11), the accuracy of this approximation depends on the recollision probability: the smaller its value is, the more accurate the approximation is. The likelihood of photons to escape the needle sample is high, and thus its recollision probability is low.

To summarize, the angular, spectral and polarimetric data convey information about properties of the needle sample surfaces (Figures 6 and 7), shoot structural organizations (Figure 8), needle sample optical properties (Figure 9) and the contribution of these components to the total radiation reflected by the samples (Figures 4 and 9). Polarization measurements are useful for remote sensing of needle absorption spectrum because they provide an estimate of the lower bound on surface reflection.

## 4. Discussion

Solar radiation reflected by a vegetation canopy and measured by a satellite-borne sensor results from photons that enter the canopy, interact with the green foliage, woody material and ground, and escape towards the sensor. The DCRF of the canopy depends on the leaf optical properties and leaf distribution in the canopy space, i.e., canopy structure, and ground reflectivity. The spectral distribution of radiation scattered by a leaf is governed by the leaf properties such as pigment concentration, chemical constituents, internal structure, and leaf-surface characteristics (Figure 1). The leaf albedo spectrum is the only optical variable that conveys information about leaf biochemistry. Radiation scattered by leaves and exiting the canopy is strongly affected by the 3D canopy structure. We use our data to quantitatively and qualitatively describe this process, with an emphasis on understanding the impact the leaf surface properties might have on inferring leaf biochemistry from satellite data. Equation (12) derived from our data coincides with solution of the 3D radiative transfer equation for vegetation canopies bounded from below by a non-reflecting surface [4]. The nesting of scales technique applied to the canopy BRF therefore will lead to a direct relationships between canopy and leaf level scattering in the form of Equation (12) in which the spectrally invariant parameters account for nested hierarchical levels present in the canopy (e.g., clumping of needle into shoots, shoots into crowns) [4, 24, 25, 27, 31, 34, 35]. Our results therefore can be directly extended to the vegetation canopies.

The structure of our samples impacts the angular distribution of specularly reflected radiation (Figure 7). Indeed, the needles in the holder form a horizontal rough surface, which mainly reflects radiation in the forward scattering directions. Needles in the *Picea koraiensis* shoot constitute a curved surface that can deflect reflected radiation from the principal plane. Needles in the *Pinus koraiensis* shoot form a sphere-like object (Figure 2), significantly reducing its magnitude in forward scattering directions, which makes the angular distribution of PDCRF more uniform (Figure 7).

Notably, the distinct structural differences in the *Picea koraiensis* needle and shoot samples (Figure 2) have not implied significant differences in their scattering coefficients (Figure 10), suggesting the recollision probabilities of the needle and shoot sample are comparable. However, for *Pinus koraiensis*, about 100 needles, 9 cm long in the shoot sample, form a sphere-like object (Figure 2), resulting in a spherical density of about eight needles per steradian. Such a low density makes the shoot very transparent and consequently the escape probability high. Figure 10b suggests that photons have a higher chance to escape the *Pinus koraiensis* shoot compared to needles densely arranged in the holder. The scattering coefficient of the *Pinus koraiensis* shoot likely provides a more accurate approximation of the needle albedo compared to its needle counterpart.

**Figure 11:**
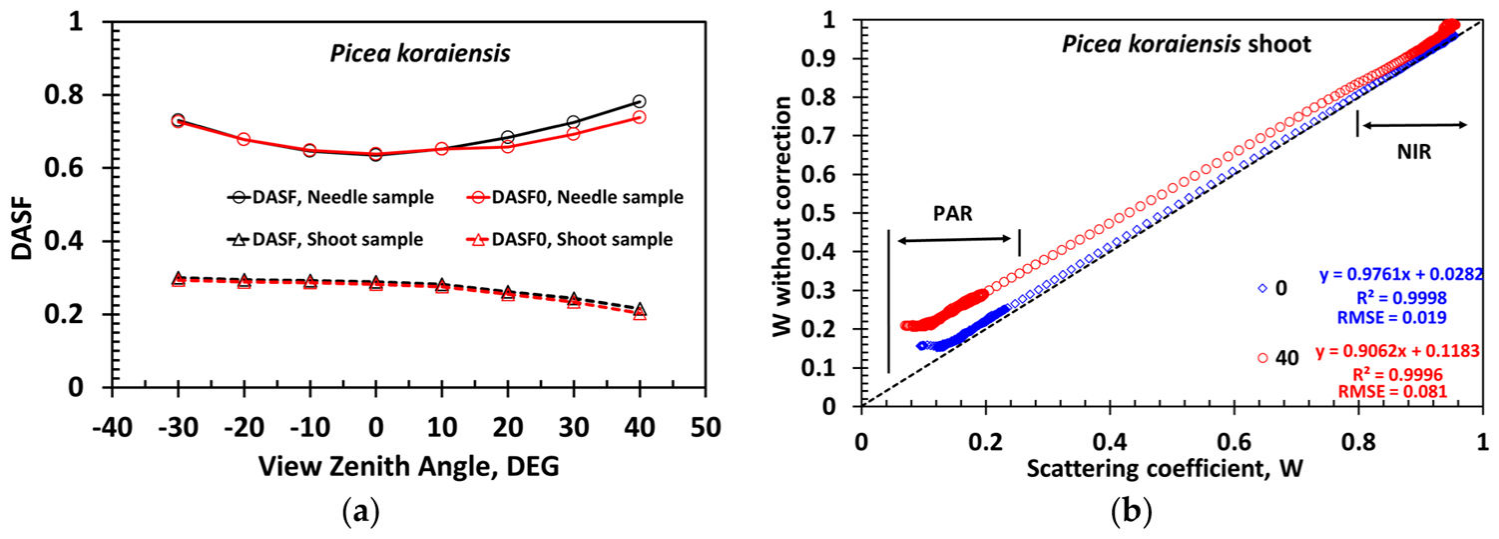
(**a**) DASF derived directly from the spectral DCRF of the *Picea koraiensis* needle (legend “DASF, Needle sample”) and shoot (legend “DASF, Shoot sample”) samples and their “true” values (from Figure 8) for needle (legend “DASF_0_, Needle sample”) and shoot (legend “DASF_0_, Shoot sample”) samples; (**b**) correlation between scattering coefficients of the *Picea koraiensis* shoot derived with (horizontal axis) and without (vertical axis) correction for the needle surface effects. In this example, relative differences are 17%–140% in blue (450–500 nm), 3%–74% red (600–650 nm), 3%–59% green (520–580 nm) and below 4% in the near infrared (800–950 nm) spectral intervals.

The DASF is a key variable that conveys information about canopy structure. It can be accurately estimated directly from the spectral DCRF without correction for the leaf surface effect (Figure 11a), indicating that the leaf surface reflected radiation (term *S_L_* in Equation (12)) minimally impacts the retrieval of canopy structural parameters, e.g., leaf area index. Ignoring this portion of reflected radiation, however, can cause an overestimation of the scattering coefficient (Figure 11b). The impact decreases from strongly (17%–140%, 450–500 nm) to weakly (<4%, 800–950 nm) absorbing wavelengths (Figure 11b). Recall that our approach provides a lower bound on the impact, i.e., a “true” impact is stronger.

Leaf surface characteristics have an impact on remote sensing of its internal constituents. The DOLP provides a direct estimate of their impact. Indeed, radiation reflected from leaf surfaces, *S_L_*, exhibits a very weak spectral dependency (Figure 6). It conveys no information about the constitution of the leaf tissue. When leaf absorption is high (i.e., *W_λ_* is low), the leaf surface reflected radiation dominates. For example, *S_L_* can be as high as 0.17 (Figures 6 and 7), whereas the diffuse component, *DCRF*_0_*_λ_* is about 0.05–0.08 at 680 nm (Figure 9). It means that only 23%–26% of the total reflected radiation carry information about leaf biochemical constituents at 680 nm. Conversely, when leaf absorption is low (i.e., *W_λ_* is high) the surface contribution is reduced compared to the diffuse component; information on the leaf inferior in the total reflected radiation is consequently increased. Polarization measurements such as from the Airborne Multiangle SpectroPolarimetric Imager (Jet Propulsion Laboratory, Pasadena, CA, USA) [36] or NASA’s planned Aerosol-Cloud-Ecosystem (ACE) Decadal Survey mission [37] can be useful to account for this source of uncertainties in inferring leaf biochemistry from spectral DCRF data.

## 5. Conclusions

The total radiation reflected by a leaf includes two components, specular and diffuse. The first component emanating from light reflected at the air-cuticle interface is polarized. The diffuse component results from photon interactions within the leaf and any large particle on the leaf surface. This portion of reflected radiation is not polarized. The purpose of our study has been to measure angular, spectral and polarimetric properties of radiation reflected by needles and shoots of two coniferous species and estimate contributions of needle surfaces, shoot structure and needle optics to the DCRF. Radiation specularly reflected from the needle sample surfaces exhibit weak spectral dependency, as expected from theory. It increases from negligible values in backscattering directions to about 17% in forward scattering directions. The shoot sample specular DCRF shows a similar behavior. Its magnitude, however, is reduced by a factor of about 10. This is attributed to the effect of the shoot structure. The fraction of specularly reflected radiation increases with increasing needle absorption and varies near the forward scattering directions between 71% at 650 nm and 13% at 820 nm.

The DASF provides critical information needed to correct the diffuse component for shoot structure effects. The removal of the effect of structural influences involves changes in the magnitude and shape of the DDCRF spectrum. The DDCRF corrected for shoot structure effects is the scattering coefficient. The canopy BRF is an explicit function of this coefficient, which, in turn, is more directly related to absorption spectra of absorbing biochemical constituents inside the needles. The specularly reflected radiation minimally impacts the retrieval of canopy structural parameters, e.g., DASF and leaf area index. Ignoring this portion of reflected radiation, however, can cause an overestimation of the scattering coefficient and consequently lowers its sensitivity to leaf biochemistry.

To summarize, the angular, spectral and polarimetric data convey information about properties of the needle surfaces, shoot structural organizations and needle optics. This information is required to retrieve the needle albedo, which is directly related to the absorption spectra of leaf biochemical constituents.

## Figures and Tables

**Table 1:** Footprint correction coefficients.

|View Zenith Angle| (°)	0	10	20	30	40
*Picea koraiensis* Needle	1.438	1.458	1.522	1.638	1.835
*Picea koraiensis* Shoot	1.064	1.065	1.065	1.067	1.069
*Pinus koraiensis* Needle	1.000	1.000	1.000	1.000	1.001
*Pinus koraiensis* Shoot	1.000	1.000	1.000	1.000	1.000

**Table 2:** View zenith angle averaged inter-calibration coefficients. Standard deviations are shown in parenthesis.

Spectral Interval (nm)	*Picea koraiensis*		*Pinus koraiensis*	

	Needle	Shoot	Needle	Shoot
450–640	0.931(0.046)	0.848(0.057)	0.876(0.050)	0.733(0.082)
650–690	0.985(0.008)	0.864(0.017)	0.979(0.033)	0.753(0.058)
700–950	1.029(0.008)	1.048(0.009)	1.039(0.012)	1.081(0.012)

## Abbreviations

The following abbreviations are used in this manuscript

ASD FS3: analytical spectral devices fieldspec 3
BRF: bidirectional reflectance factor
DCRF: directional-conical reflectance factor
DOLP: degree of linear polarization
DASF: directional area scattering factor
FOV: field of view
SZA: source zenith angle
VZA: view zenith angle
PDCRF: polarized directional-conical reflectance factor
DDCRF: diffuse directional-conical reflectance factor
RMSE: root-mean-square error

## Appendix A

The correction coefficient *k* (Ω) in a given view direction Ω is

(A1) $$k\;(\Omega )=\frac{\int_{\Delta \Omega }|\Omega \cdot \Omega^{\prime }|d\Omega^{\prime }}{\int_{\Delta \Omega_{S}}|\Omega \cdot \Omega^{\prime }|d\Omega^{\prime }}=\frac{\pi {\text{sin}}^{2}\;\left(\frac{\mathrm{FOV}}{2}\right)}{\int_{\Delta \Omega_{S}}|\Omega \cdot \Omega^{\prime }|d\Omega^{\prime }},$$

where Ω · Ω′ denotes the scalar product of unit vectors Ω and Ω′, and ΔΩ*_S_* is a solid angle of the intersection, *S* (Ω) = *A_w_* ∩ *A_f_* (Ω), of the holder window, *A_w_*, and the sensor footprint, *A_f_* (Ω) (Figure 3). The denominator in Equation (A1) can be reduced to a surface integral over *S* (Ω), i.e.,

(A2) $$\int_{\Delta \Omega_{S}}|\Omega \cdot \Omega^{\prime }|d\Omega^{\prime }=\frac{1}{R^{2}{\text{cos}}^{2}\;(\mathrm{VZA})}\int_{S(\Omega )}|\Omega \cdot \Omega^{\prime }|\cdot {|\Omega^{\prime }\cdot n|}^{3}dx^{\prime }dy^{\prime }.$$

Here, *R* = 19.8 cm is the distance between the center of the circular ring and the sensor; ***n*** = (0, 0, 1) is the outward normal to the circular ring. The direction Ω′ from a point *r*′ = (*x*′, *y*′, 0) within *S* (Ω) to the sensor location *r_S_* = (0, 0, *R*cos(*VZA*)) is Ω′ = (*r_S_* – *r*)/||*r_S_* – *r*||, where ||.|| denotes the distance between *r* and *r_S_*. The surface integral (A2) was evaluated numerically.

## Appendix B

Lewis and Disney found that the transformed leaf albedo, *ω_λ_*, can be represented as (Equations (13) and (17) in [25]),

(B1) $$\omega_{\lambda }=\omega_{0\lambda }^{k}\frac{1-p_{L}}{1-p_{L}\omega_{0\lambda }^{k}}.$$

Here, *p_L_* is the within-leaf recollision probability, and *ω*_0_*_λ_* is the reference leaf albedo. The power *k* is related to the concentrations of biochemical constituents. Both *k* and *p_L_* are wavelength independent parameters.

Let *a*_0_*_λ_* = 1 – *ω*_0_*_λ_* be the transformed leaf absorption, i.e., the probability that a photon will be absorbed by a leaf given that it interacts with internal leaf constituents. Expanding the function *f* (*x*) = (1 – *x*)*^k^* about *x* = 0 in the Taylor series and neglecting the second order term, one obtains

(B2) $$\omega_{0\lambda }^{k}={\left(1-a_{0\lambda }\right)}^{k}=1-k+k\omega_{0\lambda }+R_{1}\approx 1-k+k\omega_{0\lambda }.$$

Here, $R_{1}=\frac{k(k-1)}{2!}\;{\left(1-\theta a_{0\lambda }\right)}^{k-2}\;a_{0\lambda }^{2}$ is Maclaurin’s form of the reminder in the Taylor expansion, and θ is a number strictly between 0 and *a*_0_*_λ_*. Furthermore,

(B3) $$\frac{\omega_{0\lambda }^{k}}{\omega_{0\lambda }}=\omega_{0\lambda }^{k-1}=1-(k-1)+(k-1)\;\omega_{0\lambda }=1-q\;(k)+q\;(k)\;\omega_{0\lambda }^{k},$$

where *q* (*k*) = (*k* – 1)/*k*. Solving Equation (B3) for $\omega_{0\lambda }^{k}$, one gets,

(B4) $$\omega_{0\lambda }^{k}=\omega_{0\lambda }\;\frac{1-q\;(k)}{1-q\;(k)\;\omega_{0\lambda }}.$$

Substitution of Equation (B4) into Equation (B1) results in

(B5) $$\omega_{\lambda }=\omega_{0\lambda }\;\frac{1-p\;(k)}{1-p\;(k)\;\omega_{0\lambda }},$$

where *p* (*k*) = *q* (*k*) + *p_L_* (1 – *q* (*k*)).

Thus, for weakly absorbing wavelengths (*a*_0_*_λ_* ≪ 1), the transformed leaf albedo is related to a fixed spectrum via the spectral invariant relationship (B5). The wavelength independent coefficient *p* (*k*) depends on the concentrations of leaf absorbing constituents and mesophyll structure.

In the 710–790 nm interval, the transformed leaf albedo is mainly determined by the absorption spectra of dry matter and chlorophyll [27]. The former is flat, whereas the latter varies with wavelength. In this spectral interval, therefore, the chlorophyll absorption spectrum is a species-independent spectral curve that relates spectra of the transformed albedos. Chlorophyll absorbs little radiation in the 710–790 nm interval, i.e., *a*_0_*_λ_* ≪ 1. Maclaurin’s term in Equation (B2) can be neglected. Note that Equation (B1) is very accurate for spectral intervals where absorption of only one biochemical constituent varies with wavelength, e.g., as in the 710–790 nm interval. Additional analyses are needed if this condition is not met.
